# Studies on Clinical Features, Mechanisms, and Management of Olfactory Dysfunction Secondary to Chronic Rhinosinusitis

**DOI:** 10.3389/falgy.2022.835151

**Published:** 2022-03-04

**Authors:** Yi-Tsen Lin, Te-Huei Yeh

**Affiliations:** ^1^Department of Otolaryngology, National Taiwan University Hospital, Taipei, Taiwan; ^2^College of Medicine, National Taiwan University, Taipei, Taiwan

**Keywords:** olfactory dysfunction, smell, chronic rhinosinusitis, inflammation, cytokines

## Abstract

Chronic rhinosinusitis (CRS) is one of the most common causes of inflammation of the olfactory system, warranting investigation of the link between chronic inflammation and the loss of olfactory function. Type 2 inflammation is closely related to the clinical features and disease mechanisms of olfactory dysfunction secondary to CRS. Patients with eosinophilic CRS, aspirin-exacerbated respiratory disease, and central compartment atopic disease report increased olfactory dysfunction. Increased levels of interleukin-(IL-)2, IL-5, IL-6, IL-10, and IL-13 in the mucus from the olfactory slit have been reported to be associated with reduced olfactory test scores. The influence of several cytokines and signaling transduction pathways, including tumor necrosis factor-α, nuclear factor-κB, and c-Jun N-terminal kinases, on olfactory signal processing and neurogenesis has been demonstrated. Corticosteroids are the mainstay treatment for olfactory dysfunction secondary to CRS. Successful olfaction recovery was recently demonstrated in clinical trials of biotherapeutics, including omalizumab and dupilumab, although the treatment effect may diminish gradually after stopping the use of the medications. Future studies are required to relate the complex mechanisms underlying chronic inflammation in CRS to dysfunction of the olfactory system.

## Introduction

Olfactory dysfunction is one of the cardinal symptoms of chronic rhinosinusitis (CRS) (1–3). CRS has been reported to affect 13.4% of the American (4) and 10.9% of the European (5) general population, and up to 80% of CRS patients experience reduction or loss of smell (6), which significantly impedes quality of life (7–9). By phenotyping, CRS can be classified as chronic rhinosinusitis with nasal polyps (CRSwNP) and chronic rhinosinusitis without nasal polyps (CRSsNP). Olfactory dysfunction is observed more frequently in patients with CRSwNP (6, 10, 11), and olfaction may temporarily improve after surgery but deteriorate later (12). Olfactory dysfunction has been regarded as the consequence of obstructed air flow to the olfactory slit but increasing evidence has shown that inflammation in the olfactory neuroepithelium leads to dysfunction of the transduction of olfactory signals.

The olfactory neuroepithelium is located at the main air flow pathway in the nasal cavity and it continues with the respiratory epithelium. This spatial location makes the olfactory neuroepithelium easily susceptible to various inhaled substances, including viruses, molds, allergens, pollutants, and toxic materials. These epithelial stimulants may lead to recruitment of inflammatory cells, increased proinflammatory factors, and changes in ciliary function and secretion from goblet cells. Oral corticosteroids are the main treatment of olfactory dysfunction in patients with CRS. An initial rapid response may be followed by gradual diminishment of the treatment effect. Patients with olfactory dysfunction may eventually become corticosteroid-dependent, and long-term corticosteroid treatment may be accompanied by increasing side effects.

The critical issue is to understand the disease mechanism and find a suitable long-term treatment for olfactory dysfunction. In this review article, we present the clinical features related to olfactory dysfunction, investigate the influence of inflammation on neurogenesis and olfactory processing, and analyze the medical management of olfactory dysfunction secondary to chronic rhinosinusitis.

## Olfactory Dysfunction and Chronic Rhinosinusitis Endotypes

The emerging view is that CRS is a heterogenous syndrome resulting from a dysfunctional interaction between various environmental factors and the host immune system (3). Extensive scientific evidence has been accumulated that justifies the differentiation of CRS by recognition of more detailed endotypes, i.e., definition by the presence of particular patterns of immune cells and/or biomarkers (3). The clinical dichotomization of CRSwNP vs. CRSsNP was initially indicated by a predominance of TH1 cells in CRSsNP patients and TH2 cells and eosinophils in CRSwNP patients (13, 14). However, this definition has proven difficult to apply in East Asia where a neutrophilic type of inflammation with involvement of other T-cell subsets, such as TH1 and TH17 cells, has been observed aside from eosinophil-dominant inflammation (15–18). The EPOS2020 categorizes primary CRS by endotype dominance into type 2 or non-type 2 (3). Type 2 inflammation is characterized by the presence of increased levels of cytokines interleukin-(IL-)4, IL-5, and IL-13, as well as activation and recruitment of eosinophils and mast cells.

The risk factors for olfactory dysfunction differed between CRS endotypes, and CRS patients with type 2 inflammation endotype reported loss or reduction of olfaction more frequently than those with non-type 2 CRS (19). Esoinophilic CRS is the most dominant type of type 2 CRS, and the association of esosinophilic CRS and olfactory dysfunction has been well-recognized (18, 20). The most apparent difference in computed tomography (CT) images of eosinophilic CRS compared to non-eosinophilic CRS images is an ethmoid sinus predominance pattern (21). CT images of the early stage of eosinophilic CRS show opacification of the posterior ethmoid sinus and the olfactory cleft (18). Mori et al. (19) identified olfactory cleft polyps, current smoking, serum IgE ≥400 IU/ml, ethmoid opacification, and asthma as independent risk factors for olfactory dysfunction in eosinophilic CRS. In non-eosinophilic CRS, only ethmoid opacification and olfactory cleft polyps were identified as independent risk factors for olfactory dysfunction. Aspirin-exacerbated respiratory disease (AERD) has also been identified as one of the independent factors of olfactory dysfunction in CRS patients (22, 23).

Central compartment atopic disease (CCAD) is another CRS subtypes of type 2 CRS. DelGaudio et al. (24) were the first to define CCAD as one of the subtypes of CRS in 2017 and associate CCAD with inhalant allergen sensitization. CCAD is characterized as an inflammation and edematous change of the central sinonasal compartment, including the middle turbinate, superior turbinate, and posteriosuperior nasal septum (24, 25). CCAD presents as a polypoid edema of the middle turbinate on endoscopic examination. A centrally limited sinus inflammation entity on the CT scan has been defined as having normal sinus mucosal or mucosal thickening involving only the floor or medial wall of the ethmoid sinuses (25, 26). We previously found that central-compartment-type CRS represented an eosinophilic/type 2 inflammation endotype, with elevated expression of IL-5 and IL-13 in the sinonasal tissues, and patients with this central-compartment subtype of CRS had more smell problems as major symptoms than patients with other CRS subtypes (27).

## Mechanisms of Olfactory Dysfunction Secondary to CRS

**Figure 1:**
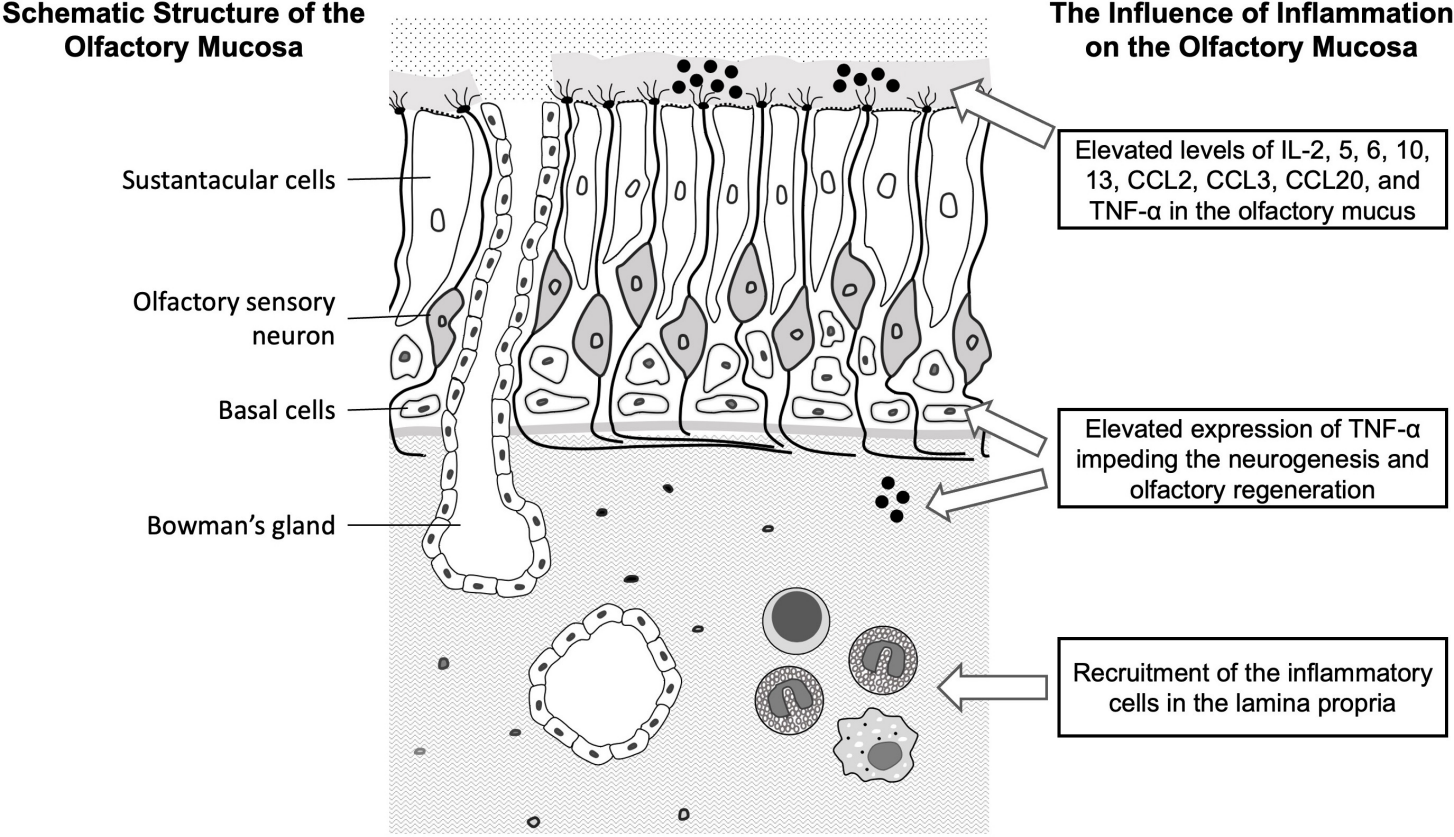
The illustration of the structure of the olfactory mucosa and the known inflammatory mechanisms leading to olfactory dysfunction. IL, interleukin; CCL, C-C motif chemokine ligand; TNF, tumor necrosis factor.

With advances in immunologic and histopathological studies, the rationale of olfactory dysfunction secondary to CRS is regarded not only as diminished access of odorant molecules to the neuroepithelium (conduction disorder) but also direct effects on the olfactory mucosa (sensory disorder) (10). Olfactory dysfunction due to chronic rhinosinusitis is relatively reversible compared to other causes. As short-term anti-inflammatory treatment can generally render rapid regain of olfactory function, the mechanism of olfactory dysfunction may be reversible after elimination of inflammation. The schematic diagram of the olfactory mucosa and the current evidence of the impact of inflammation on the peripheral olfactory system were illustrated in the Figure 1, and the possible mechanisms and molecules involved in the development of olfactory dysfunction were summarized in the Table 1.

**Table 1:** Summary of the studies on the mechanisms of olfactory dysfunction in CRS.

**Study**	**Methodology**	**Measure of olfaction**	**Main outcomes**
**Histopathological study**			
Kern (28)	Olfactory epithelium	UPSIT	Infiltration of lymphocytes, macrophages, and eosinophils in the lamina propria of the olfactory epithelium in patients of CRS with olfactory dysfunction
Yee et al. (29)	Olfactory epithelium	Olfactory threshold task of phenylethyl alcohol	Increased erosion of the olfactory epithelium and eosinophils infiltrating the olfactory epithelium in patients of CRS with olfactory dysfunction
Hauser et al. (30)	Ethmoid mucosa	40-item smell identification test	An association of tissue eosinophilia in the ethmoid sinuses and olfactory dysfunction
Lavin et al. (31)	Superior turbinate	UPSIT	An association of tissue eosinophilia in the superior turbinate and olfactory dysfunction
Wu et at. (32)	Superior turbinate	Sniffin' Sticks	An association of tissue eosinophilia in the superior turbinate and post-operative olfactory dysfunction
**Association study**			
Schlosser et al. (33)	Measurement of biomarkers in collected olfactory cleft mucus	Sniffin' Sticks	A correlation of IL-5 levels and olfactory dysfunction in both CRSwNP and CRSsNP
Wu et al. (34)	Measurement of biomarkers in collected olfactory and middle meatal mucus	Smell Identification Test	Elevated levels of IL-2, IL-5, IL-6, IL-10, and IL-13 associated with reduced test scores for smell identification, especially in CRSwNP patients
Morse et al. (22)	Measurement of biomarkers in collected middle meatal mucus	Smell Identification Test	An association between IL-2, IL-5 and IL-13, and olfaction
Soler et al. (35)	Measurement of biomarkers in collected olfactory cleft mucus	Questionnaire of Olfactory Dysfunction	An association and elevations in TNF-α, IL-6, CCL2, CCL3, and CCL20 with lowest olfactory scores in clusters dominated by type 2 biomarkers
**Animal study**			
Turner et al. (36)	Unilateral olfactory bulbectomy on IOI mice^a^	IHC study of the olfactory epithelium	Suppression of olfactory regeneration by TNF-α
Sultan et al. (37)	Systemic corticosteroid treatment on IOI mice^a^	IHC study of the olfactory epithelium; EOG	TNF-α causing physiologic dysfunction of olfactory neurons; prednisolone preventing neuronal loss and olfactory dysfunction by diminishing the subepithelial inflammation
Pozharskaya et al. (38)	*TNFR2* knockout in IOI background mice^a^	IHC study of the olfactory epithelium; EOG	TNFR2 mediating neuronal proliferation and death but not TNF-α-induced dysfunction of mature olfactory sensory neurons
Sousa Garcia et al. (39)	*TNFR1* knockout in IOI^a^ background and allergen-induced inflammation mice	IHC study of the olfactory epithelium; EOG	TNFR1 regulating TNF-α-induced inflammation and reduces allergen-induced inflammation

a *Inducible olfactory inflammation mice is a genetic model of olfactory inflammation by temporally controlled induction of TNF-α by olfactory sustentabular cells*.

*CRS, chronic rhinosinusitis; UPSIT, University of Pennsylvania Smell Identification Test; IL, interleukin; CRSwNP, chronic rhinosinusitis with nasal polyps; CRSsNP, chronic rhinosinusitis without nasal polyps; TNF, tumor necrosis factor; CCL, C-C motif chemokine ligand; IOI, inducible olfactory inflammation; IHC, immunohistochemical; EOG, electro-olfactogram*.

### Change in Histopathologic Images of the Olfactory Epithelium in CRS

The respiratory mucosa is pseudostratified and ciliated with goblet cells, a highly vascular lamina propria and a thick basement membrane, whereas the olfactory mucosa is characterized by irregular cilia, a cellular lamina propria, Bowman's glands, and a thin basement membrane. The olfactory neuroepithelium contains three major cell types of the peripheral sensory system, including olfactory sensory neurons (OSNs), sustentacular cells, and basal cells (Figure 1). Sustentacular cells enwrap olfactory sensory neurons to maintain the integrity and function of OSNs. Basal cells are located along the basement membrane and capable of replenishing OSNs to maintain ongoing neurogenesis during adult life. OSNs and their progenitors are particularly susceptible to local immune mediators in the setting of rhinosinusitis (40, 41).

As we observed compromised integrity of the epithelium and infiltrating immune cells in the respiratory epithelium in cases of CRS, similar histopathology in the olfactory neuroepithelium may account for the dysfunction of the peripheral olfactory system (28). Yee et al. (29) studied the neuropathology of the olfactory mucosa in CRS and found a significant decrease in the percentage of normal olfactory neuroepithelium, a reduction of mature OSNs in CRS biopsy specimens, and a variety of epithelial changes, including intermixing of goblet cells, metaplasia to squamous-like cells, and erosion of the olfactory neuroepithelium. With continued inflammation in the olfactory mucosa, an abnormal epithelium and infiltration of lymphocytes, macrophages, and eosinophils in the lamina propria were identified (28). Eosinophilic infiltration may play a significant role in cases of olfactory disability, and CRS patients, especially those with nasal polyps, presenting with anosmia had the greatest amount of epithelial erosion and the highest density of eosinophil infiltrations (30). Moreover, increased eosinophils in the superior turbinate have been correlated with the degree of olfactory dysfunction and olfactory decline after sinus surgery (31, 32). Inflammation of the epithelium can affect olfactory neurogenesis, differentiation, and maturation of OSNs.

### The Role of Cytokines and Immunologic Biomarkers

Researchers have worked on identifying types of cytokines or biomarkers in the olfactory neuroepithelium, and increased levels of several cytokines in the olfactory cleft have been correlated with olfactory dysfunction in CRS patients.

In a study by Henkin et al., (42) increased levels of IL-6 in nasal mucus, saliva, and plasma were reported in hyposmia patients due to various causes. IL-6 is a proinflammatory cytokine, and inflammation was proposed to play a role in the biochemical pathological process underlying hyposmia. Schlosser et al. (33) corrected the mucus defect in olfactory clefts by inserting a polyurethane sponge into each olfactory cleft under endoscopic guidance and correlated the threshold discrimination identification score of the Sniffin' Sticks test with the levels of secreted mediators. IL-5 levels were found to be inversely correlated with olfactory test scores in both CRSwNP and CRSsNP. In another study by Wu et al., mucus was collected from both the olfactory cleft and middle meatus of CRSwNP, CRSsNP, and control subjects to compare the expression of cytokines/chemokines and olfactory function. Elevated levels of IL-2, IL-5, IL-6, IL-10, and IL-13 were associated with reduced test scores for smell identification, especially in CRSwNP patients. A strength of the aforementioned study was that cytokine levels in the middle meatus were demonstrated to be compatible with those in the olfactory cleft, which has considerable clinical significance because secretion in the middle meatus is more applicable in clinical settings than secretion in the olfactory cleft (34). The same research group applied a hierarchal cluster analysis and machine learning algorithms to data from 110 patients to characterize inflammatory patterns and correlated these patterns with smell identification scores. Olfaction was found to be strongly correlated with levels of cytokines IL-5 and IL-13, whereas the mucus IL-12 levels, CT score, and AERD were independently associated with olfactory dysfunction in CRS patients (22). In another cluster analysis of olfactory cleft mucus biomarkers, including cytokines, chemokines, and growth factors, Soler et al. demonstrated that clusters dominated by IL-4, IL-5, IL-13, and IgE were associated with lowest olfactory scores and elevations in tumor necrosis factor-α (TNF-α), IL-6, and chemokines that promote monocyte/macrophage recruitment (C-C motif chemokine ligand [CCL]2, CCL3, CCL20) (35).

### The Effect of Inflammation on Neurogenesis

Several cytokines have neurotoxic potential, and the effects of cytokines may mediate OSN function and regeneration. OSNs of abnormal morphologies and potential functional defects have been reported in CRS patients, along with increased numbers of immature neurons (43). However, the effect of cellular and molecular pathways and underlying mechanisms of CRS-associated inflammation on the function of the peripheral olfactory system remain incompletely understood. This gap in our knowledge may be due to the limited accessibility of human olfactory tissue and the difficulty of maintaining human olfactory neurons in standard cell cultures. Holbrook et al. (44) performed an autopsy study and used immunohistochemical analysis to compare the molecular phenotypes of olfactory epithelial cells between rodents and humans. The immunostaining patterns showed there were two distinct basal cell types, horizontal and globose, in both humans and rodents, and this similarity between experimental animals and humans could shed light on olfactory pathophysiology. The effect of inflammatory mediators on the apoptosis, differentiation, and proliferation of OSNs in animal models has been investigated.

TNF-α is a pleiotropic cytokine that has been universally associated with CRS, regardless of subtype or etiology (45). TNF-α plays a role in antigen-specific immunoglobulin E (IgE) production and Th2 cytokine production and modulates the migration of Th2 cells to inflammation sites (46–50). Lane et al. investigated a transgenic mouse model expressing TNF-α by sustentacular cells in the olfactory epithelium: TNF-α was found to directly affect olfactory neuron function and neuroepithelial regeneration, and the downstream mediators following infiltration of inflammatory cells contributed to histological damage to the olfactory neuroepithelium (36, 38, 40, 51). Sousa Garcia et al. demonstrated that genetic deletion of *TNFR1* (tumor necrosis factor α receptor 1) in inducible olfactory and allergen-induced inflammation models prevented histological damage, reduced eosinophilic infiltration, and preserved the neuronal layer thickness. *TNFR1* may be crucial in the development of inflammation-associated olfactory dysfunction (39). The acute inflammatory response may promote regeneration of olfactory neuroepithelium through the nuclear factor-κB (NF-κB) pathway (52), and c-Jun N-terminal kinases (JNK), the principal signaling molecules involved in the TNF-α apoptotic pathway, were found to be activated in neuroinflammation (53). The expression of TNF-α in the olfactory neuroepithelium is critical in the pathogenesis of olfactory dysfunction.

Rouyar et al. investigated the impact of type 2/Th2-driven inflammation on olfactory function in a mouse model of ECRS sensitized to house dust mites and *Staphylococcus aureus* enterotoxin B. The expression of IL-4, IL-5, IL-13 and total IgE in the olfactory epithelium was significantly increased in the group treated with house dust mites, and IL-13Rα1 and IL-4Rα mRNAs was detected in mature OSNs, globose basal cells, and horizontal basal cells, which are involved in OSN renewal. The transcriptomic and histology markers revealed a decrease in the number of immature OSNs that did not affect the sense of smell, as measured by electroolfactogram and animal behavioral food tests (54). The roles of the IL-4 and IL-13 pathways and their possible regulatory impacts on neurogenesis and homeostasis of olfactory neurons have not been determined. According to the olfactory vector hypothesis, some neurological disorders may be caused or accelerated by agents entering the central nervous system through the olfactory bulb *via* the olfactory mucosa (55). Mori et al. suggested that IL-4 and IL-13 cytokines may contribute to pathological mechanisms leading to the loss of dopaminergic neurons in Parkinson's disease (56). Further studies on neuroinflammation and damage in other brain regions could provide novel insights into the olfactory vector hypothesis and facilitate application of this hypothesis to the pathogenesis of possible central olfactory disorders (41).

## Treatment of Olfactory Dysfunction in Chronic Rhinosinusitis

Despite limited effective treatment choices for olfactory dysfunction, olfactory dysfunction related to CRS is regarded as a treatable trait. In contrast to other non-CRS-related olfactory dysfunctions, corticosteroids are an effective mainstay treatment for the management of smell problems secondary to CRS (57). Endoscopic sinus surgery can aid the recovery of olfactory function (58). Sinus surgery can remove the diseased tissues and improve the nasal ventilation, and it has been reported that current septoplasty can increase the likelihood of achieving normal olfaction (59). A meta-analysis reported by Zhao et al. (60) demonstrated that endoscopic sinus surgery may be beneficial olfactory dysfunction The olfactory function, assessed by Sniffin' Sticks total score, discrimination score and identification score, University of Pennsylvania Smell Identification Test, and Visual Analougue Scale, improved in the patients of CRSwNP. However, the results of olfaction function after surgery were reported to be inconsistent in the CRSsNP and non-classified CRS patients. The inflammatory natures of CRS are decisive in the olfactory outcomes after the surgery. Comparing to the relatively well-sustained olfaction in non-eosinophilic CRS patients, the improvement of olfaction deteriorated with time among eosinophilic CRS patients (61). Administration of adjuvant medical therapy post-operatively may aid the continued recovery of olfactory function.

Biotherapeutic agents targeting type 2 inflammation were recently introduced into the treatment of CRS (62). The biologics dupilumab (63–65), omalizumab (66, 67), and mepolizumab (68) have been shown to improve olfaction based on symptom scores and olfaction tests. Dupilumab, blocking the shared receptor component of IL-4 and IL-13, can even improve olfaction function in patients who previously underwent more than 3 surgeries (69, 70). Successful treatment with biologics may imply that type 2 inflammation plays a role in the disease mechanism of olfactory dysfunction in CRS. A transcriptomic study of respiratory epithelial, immune, and stromal cell types and subsets in the ethmoid sinus of patients with CRS showed that epithelial stem cells may contribute to the persistence of inflammatory disease by serving as repositories for allergic memories, and a shift from interferon-α (IFN-α)/IFN-γ-induced genes to IL-4/IL-13-induced genes was demonstrated to be correlated with disease severity. A comparison of epithelial cells scraped before and 6 weeks after dupilumab treatment showed that this IL-4 receptor α-subunit blocker can modify basal and secretory cell states *in vivo* (71). The role of type 2 inflammation-mediated barrier dysfunction in the olfactory neuroepithelium remains to be determined.

## Conclusion

Evidence from clinical features, experimental investigations, and treatment responses shows that type 2 inflammation may play an important role in olfactory dysfunction secondary to CRS. In-depth research on the link between the olfactory transduction pathway and inflammation is warranted. The olfactory system and frontline of the respiratory immune system share the same airway passage in the nose, and understanding the impact of epithelial barrier dysfunction, localized inflammation, and aggregation of immunocytes on the olfactory neuroepithelium may shed light on the management of olfactory dysfunction. An effective and sustained treatment for patients with olfactory dysfunction secondary to CRS can significantly improve patients' quality of life.

## Author Contributions

All authors listed have made a substantial, direct, and intellectual contribution to the work and approved it for publication.

## Funding

This study was supported by grants #110-S5159 and #111-X0039 from National Taiwan University Hospital.

## Conflict of Interest

The authors declare that the research was conducted in the absence of any commercial or financial relationships that could be construed as a potential conflict of interest.

## Publisher's Note

All claims expressed in this article are solely those of the authors and do not necessarily represent those of their affiliated organizations, or those of the publisher, the editors and the reviewers. Any product that may be evaluated in this article, or claim that may be made by its manufacturer, is not guaranteed or endorsed by the publisher.
